# Advances in Biodegradable Polymers and Biomaterials for Medical Applications—A Review

**DOI:** 10.3390/molecules28176213

**Published:** 2023-08-24

**Authors:** Małgorzata Oleksy, Klaudia Dynarowicz, David Aebisher

**Affiliations:** 1 Students English Division Science Club, Medical College of the University of Rzeszów, 35-959 Rzeszów, Poland; mo121935@stud.ur.edu.pl; 2 Center for Innovative Research in Medical and Natural Sciences, Medical College of the University of Rzeszów, 35-310 Rzeszów, Poland; kdynarowicz@ur.edu.pl; 3 Department of Photomedicine and Physical Chemistry, Medical College of the University of Rzeszów, 35-959 Rzeszów, Poland

**Keywords:** biodegradable polymers, biomaterials, applications of biomaterials in medicine, applications of biodegradable polymers in medicine

## Abstract

The introduction of new materials for the production of various types of constructs that can connect directly to tissues has enabled the development of such fields of science as medicine, tissue, and regenerative engineering. The implementation of these types of materials, called biomaterials, has contributed to a significant improvement in the quality of human life in terms of health. This is due to the constantly growing availability of new implants, prostheses, tools, and surgical equipment, which, thanks to their specific features such as biocompatibility, appropriate mechanical properties, ease of sterilization, and high porosity, ensure an improvement of living. Biodegradation ensures, among other things, the ideal rate of development for regenerated tissue. Current tissue engineering and regenerative medicine strategies aim to restore the function of damaged tissues. The current gold standard is autografts (using the patient’s tissue to accelerate healing), but limitations such as limited procurement of certain tissues, long operative time, and donor site morbidity have warranted the search for alternative options. The use of biomaterials for this purpose is an attractive option and the number of biomaterials being developed and tested is growing rapidly.

## 1. Introduction

According to the European Society of Biomaterials, a biomaterial is defined as a substance other than a drug or a combination of several substances (of synthetic or natural origin) that can be used at any time as part of an organ, to treat, enhance or restore body functions [1,2,3,4]. It is a group of materials with different compositions, structures, and properties that affect their acceptance by the body and the ability to connect and regenerate human tissues.

Biomaterials play a major role in the estimated USD 400 billion global medical device market however, biomaterials are foreign bodies, and therefore adverse host reactions pose a fundamental challenge that can drastically reduce the quality of life of patients, which explains their current poor clinical use [5,6,7]. These side effects often interfere with the healing process, causing extreme pain to the patient, excessive inflammation, and tissue destruction, and can lead to rejection. The lack of a detailed understanding of the interactions between biomaterials and the immune system is a major obstacle to the development of effective biomaterial-based therapies and tissue engineering approaches [5,6,7]. Therefore, one of the main challenges for the biomaterials community is to design multifunctional materials with functions such as degradability [8,9,10,11], the ability to control drug release [12], or sensitivity to stimuli [13], while addressing ways to overcome immune barriers [14], inflammation [15], and endotoxin contamination [16].

Since ancient times, biomaterials have been used as raw materials for the production of implants and prostheses. However, the use of these materials drastically accelerated after World War II [17,18,19]. The global biomaterials market is estimated to reach USD 47.5 billion by 2025 from USD 35.5 billion in 2020, with a compound annual growth rate (CAGR) of 6.0% at the time of the forecast [20]. This is probably due to the growing development of new biomaterials, the demand for medical implants, and the growing incidence of cardiovascular diseases, which translates into increasing subsidies under the health policy of many countries around the world. In addition, high demand for this type of material in plastic surgery is expected, which will also translate into wider use of this type of material [20,21,22,23].

Commonly, therapeutic biomaterials can be divided into two main categories:

1. Living or once living material of animal or human origin;

2. Other materials, including materials of plant origin and synthetic materials and their composites, which are biocompatible and can be used for tissue regeneration [24,25].

**Figure 1:**
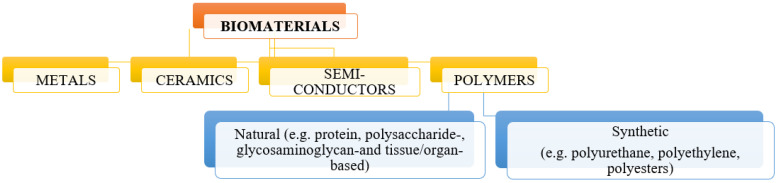
Division of biomaterials according to their properties.

Biomedical materials can be divided according to several criteria, including their properties, behavior in the body, and duration of their safe use for the patient’s body [26]. According to the division referring to the various properties of biomaterials, three basic groups can be distinguished: metal biomaterials, ceramic biomaterials, and polymer biomaterials (Figure 1). These materials, due to their interesting and different properties, can be used to create bio-composite materials, which are promising materials for use in medicine and tissue engineering.

Biomaterials can be found in every field of medicine in various forms, including various types of implants (e.g., biomechanical), artificial organs or their parts, drug carriers, or tissue joining devices (e.g., surgical sutures) [4]. Processing techniques such as foaming, pressing or casting are usually used to produce these types of products. These techniques, however, are expensive, require a long production time, and the products obtained are often not adapted to the needs of the patient. Today, most of the production of implants is based on additive manufacturing methods, due to the economic approach to production and the possibility of producing much more complex structures. One of the most important techniques of additive manufacturing is 3D printing, which, thanks to modern computer-aided techniques, such as CAD/CAM, makes it possible to obtain a component product that is ideally suited to the patient’s needs, but also to avoid human errors during manufacture [27,28].

The primary role of biomaterials in tissue engineering is to provide temporary mechanical support and mass transport to promote cell adhesion, proliferation, and differentiation, and to control the size and shape of the regenerated tissue [29]. In addition, biomaterials, usually described as scaffolds, can exhibit physical and chemical signals with spatio-temporal accuracy that are of great importance for modulating cell efficiency and function and guiding proper tissue regeneration [30]. Identifying the right biomaterials for cell accommodation and mass transport is a key step in any tissue engineering project. So far, there are many possibilities for designing a specific biomaterial that is ultimately to be used as a matrix, including (1) natural biomaterials, and (2) synthetic biomaterials and composites composed of many types/classes of materials [31,32].

## 2. A Review of Biomaterials

### 2.1. Metallic Biomaterials

Metallic biomaterials are the most commonly used group of biomaterials. This is due to the wide use of this material in medicine, especially in prosthetics and surgery, which is due to the fact that metal implants have a long lifetime of about twenty years [27].

Metals used as biomaterials should be mainly non-toxic and bio-tolerant. Thanks to features such as excellent electrical and thermal conductivity and excellent mechanical properties (such as good strength under static and dynamic loads), these materials are used in every branch of medicine. They are used as passive substitutes during fracture healing, spine fixation devices, and many kinds of implants, e.g., hip and knee joints. A careful choice of biomaterials used in vivo should be made due to possible consequence of negative effects of corrosion is the spontaneous disintegration of the implant material, its weakening, and the harmful effect of corrosion products on the surrounding tissues and organs described in some studies [4,33,34].

**Figure 2:**
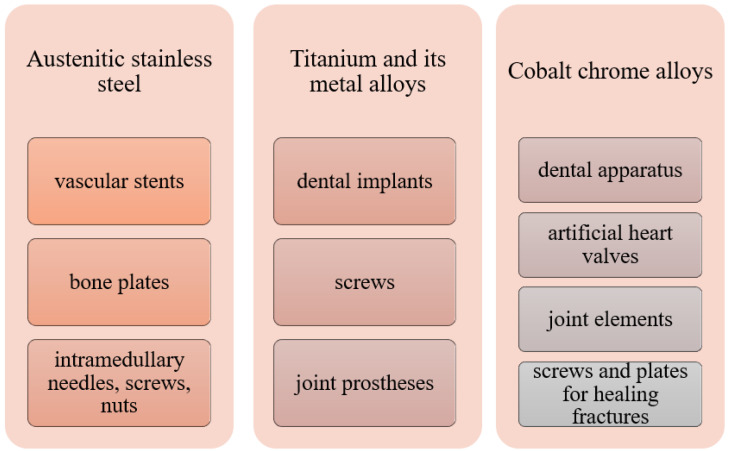
Medical use of selected metallic biomaterials [4,35].

Among the most commonly used metallic materials used in medicine, we can distinguish austenitic steels, titanium and its alloys, an alloy of cobalt with chromium or molybdenum or nickel, and precious metals. Figure 2 shows some applications of metallic biomaterials.

A very interesting group of metallic materials are currently biodegradable materials, on which numerous scientific studies are being carried out. Biodegradable metal scaffolds made of these materials show similar mechanical properties to human bone. In addition, it has been shown that biodegradable metals may have better mechanical properties compared to biodegradable polymers. Such materials include porous magnesium or iron alloys. Research has recently been carried out on the introduction of magnesium into the polymer matrix. The study showed that magnesium would provide higher mechanical strength and fracture toughness, and the polymer would prevent premature degradation of the composite [36].

Currently, along with the growing interest in nanotechnology, materials used in the manufacture of medical equipment have begun to be modified. The use of metallic nanoparticles, including titanium, zirconium, silver, gold, zinc, or copper, ensured improved antibacterial, mechanical, and regenerative properties. The introduction of metal nanoparticles into bone scaffolds allowed not only the improvement of the mechanical properties but also to increase the cell adhesion of osteoblasts and chondrocytes. Moreover, thanks to the use of nanomaterials, implants capable of releasing drugs can be developed while maintaining therapeutic requirements such as drug load, dosage, and release rate [27,36].

### 2.2. Ceramic Biomaterials

Ceramic materials are polycrystalline materials with a number of interesting features that make them desirable materials in medicine and tissue engineering. These materials, in addition to corrosion resistance in the environment of tissues and body fluids, show a very high bio-tolerance in the body [4]. The ability to bond ceramics with tissues is affected by such features as composition, crystallinity, particle size, and porosity, which can be controlled during the processing of bio-ceramics [36]. These materials consist mainly of metallic and non-metallic elements connected by strong covalent and/or ionic bonds [9]. The presence of these types of bonds makes these materials hard, brittle, and stiff; they have low resistance to loads and dynamic bending, and they also have low electrical and thermal conductivity. Currently, thanks to the progress of science and technology, ceramics, and mainly its composites, can be used not only as medical devices to strengthen or renew various parts of the body but also as a material in controlled drug release, gene and cancer therapies.

#### Bio-Ceramic Biomaterials

There are three main types of bio-ceramics:

- Resorbable or biodegradable (non-inert),
- Surface-reactive or bioactive (semi-inert),
- Non-absorbable (inert).

Bio-ceramic materials when resorbed into the body include materials that, when placed in the human body, begin to degrade into harmless compounds and can replace emerging tissue. These materials have a chemical and mineralogical composition similar to the inorganic substance of bones and teeth. They do not produce any toxic or carcinogenic reactions but show high biocompatibility [33]. The main representatives of this class are calcium phosphates and calcium aluminate ceramics.

Bioactive ceramics mainly include hydroxyapatite, bio-glasses, and bio-glass ceramics. These materials interact with the surrounding bone or soft tissue due to an ion exchange reaction between the bioactive implant and the surrounding body fluids. This reaction causes the formation of a biologically active layer of carbonate apatite (CHAp) on the implant, which is chemically and crystallographically equivalent to the mineral phase in the bone [21].

Ceramic inert materials show only minimal interaction with the surrounding tissue. The disadvantage of the above-mentioned materials is the difficulty with their fixation in living tissue, which is possible through the use of different cements. In order to eliminate the problem associated with the fixation of these types of implants, bio-intrinsic porous materials were used, which include, among others: aluminum oxide, pyrolytic carbon, silicon nitride or oxynitride, silicon carbide, zirconium, titanium or magnesium oxides [4]. The use of these types of materials not only improved the connection between the implant and the tissue, but also due to better mechanical properties, the life of the implant was significantly extended.

**Figure 3:**
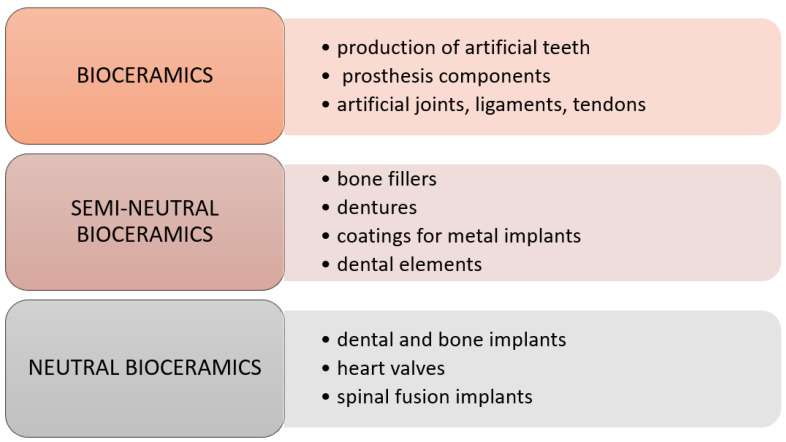
Medical application of ceramic biomaterials [9].

Bio-ceramic materials are mainly used in orthopedics and dentistry. These materials are used to produce various types of prostheses and artificial bone elements, e.g., auditory, nasal, and orbital bones, but also as bone cement or sealant of nerve canals in dentistry. Selected applications of individual types of bio-ceramics are shown in Figure 3.

### 2.3. Polymer Biomaterials

Polymers are a very interesting and constantly developing group of materials. These materials, due to their favorable and different mechanical and physicochemical properties, have become very promising materials in medicine and related fields. In addition, the main advantages of polymeric materials are ease of processing, low cost of production, and the possibility of modification. However, these materials have a number of disadvantages, including absorbing water and protein in the human body, and their surfaces are easily contaminated and difficult to sterilize [35]. Therefore, before their potential use in medicine, they undergo a number of tests and modifications.

**Figure 4:**
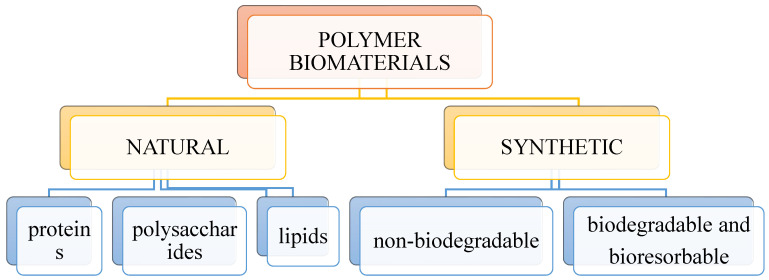
Division of polymeric biomaterials.

Biomedical polymers, as well as any other types of polymers, can be divided according to many criteria. Due to their origin, two groups of biopolymers can be distinguished: polymers of natural and synthetic origin, as shown in Figure 4.

A key subset of polymeric biomaterials is biomaterials of natural origin, which can be successfully used as tissue engineering templates due to their bioactivity, biocompatibility, controlled degradation, and mechanical kinetics, and their intrinsic structural similarity to the extracellular matrix (ECM) of native tissue. Most often, natural biopolymers are processed using environmentally friendly water-based methods. Moreover, when used in biological systems, they do not release cytotoxic products during the degradation process. The speed of the process can be adjusted by changing the starting formula and/or processing conditions [37]. The advantage of natural biomaterials is their inherent ability to promote biological recognition, which can positively support cell adhesion and function [38]. In addition, in nature, helical macromolecules such as collagen, cellulose, and chitin are of great importance for the morphogenesis and functionality of many different materials with a hierarchical structure [39].

Naturally occurring polymers are obtained in living organisms as structural components of tissues. These materials include proteins such as collagen and silk, and polysaccharides such as cellulose or chitin. Natural biomedical polymers have unique biophysical and biochemical properties. Features such as biocompatibility, biodegradability, higher adsorption of body fluids, the ability to form a gel, non-toxicity, non-immunogenicity and antifungal, antibacterial, and anticancer properties [40].

#### 2.3.1. Biomaterials of Natural Origin

Biomaterials of natural origin can usually be divided into two groups. One is protein-based biomaterials (e.g., collagen, elastin, fibrin, keratin, silk, and gelatin), which are typically derived from animal and human sources and include bioactive molecules that mimic the extracellular environment, while polysaccharide-based biomaterials are mainly derived from algae such as in the case of agar and alginate, or from microbial sources, as in the case of dextran and its derivatives [41,42,43]. Another class of natural biomaterials is referred to as decellularized tissue-derived biomaterials, which result from the elimination of all cellular and nuclear materials from native tissues/organs, such as decellularized dermis, heart valves, blood vessels, small intestinal submucosa, and liver. These decellularized tissue-derived biomaterials contain a wide variety of organic and/or inorganic components. Some natural polymers also contain surface ligands or motifs required for cell adhesion and proliferation. In particular, cell adhesion and subsequent cell activity are mediated by specific integrin–ligand interactions between cells and their surrounding extracellular matrix (ECM) [44].

Due to the key advantage of these materials in promoting cell attachment, proliferation, and differentiation, natural polymers have been extensively studied in the development of tissue engineering templates, often in combination with molecular and mechanical signals for applications ranging from tissue repair to functional organs [45]. In therapeutic applications, these polymers are generally processed for implantation as porous scaffolds, hydrogels, particulates, or thin membranes, and typically are enzymatically degraded to non-toxic end products in vivo. While the degradation kinetics of these biomaterials may not be easy to control or predict, they are still effective if local, short-term responsive action is sufficient. In addition, special forms of natural polymers (e.g., injectable hydrogel) can be administered non-invasively to the target site of tissue damage [43,46,47,48].

The disadvantages of biomaterials of natural origin are generally poor mechanical strength and inconsistency in composition and properties that are associated with serial production due to their origin from living organisms [49]. To overcome these limitations, recent advances in the redesign and fabrication of tissue-engineered templates have led to a paradigm shift towards the development of biomimetic scaffolds that contain ligands that mimic native ECM. These scaffolds are often used in vitro as analogs of natural ECM to facilitate studies of cell–ECM interaction and other complex processes [50,51]. Another problem with bio-based polymeric materials is the variability associated with the production of the materials and the potential, albeit small, of the materials to elicit an immune response [52]. The structural compositions of the most common proteins used in materials (i.e., silk, elastin, resilin, collagen, and keratin) all have exceptional mechanical strength and elasticity. The unique properties of these proteins are inherently linked to their composition, typically multiple tandem repeats of short amino acid sequences [53]. The advantage of creating hydrogels from proteins is that function can be readily introduced into the structural matrix [54].

Up to date the structure of chitosan allowed for many functional modifications and broad application in biotechnology and medicine. The newly obtained selenium-containing cationic chitin and chitosan derivatives exhibit a high transfection activity and are promising gene delivery vectors [55,56]. There are several antimicrobial dressings and drug delivery vehicles using chitosan and chitosan derivatives are approved by the FDA [57]. Therefore, chemical modification of chitosan can increase the solubility of chitosan in a water medium and enhance chitosan’s antimicrobial properties. Chitosan serves as an antimicrobial agent, a drug delivery carrier for antimicrobial agents, and prebiotics to enhance colonization resistance against pathogens. Chitosan can conjugate with other reactive components as antimicrobial agents.

Among the biopolymers, chitosan is one of the most popular due to its biocompatibility, biodegradability, antibacterial properties, and ease of use. Due to its ability to inhibit Gram-negative and Gram-positive bacteria, yeasts, and food-borne filamentous fungi, chitosan is a suitable biopolymer for the development of food packaging [58]. In the last few decades studies devoted to the elaboration of antibacterial films for biomedical food applications. Such films can be used in medicine for the treatment and healing of wounds, burns, caries, bones, and mucosal injuries. In addition, such films can be loaded with drugs and act as systems for prolonged drug release at the sites of pathological processes. Chitosan oligosaccharide sensitizes multidrug-resistant Staphylococcus aureus to antibiotic formulations by electrostatically interacting with multidrug efflux pumps [59]. Many reviews [60,61,62,63] showed the role of chitosan in an evaluation of antimicrobial and antibacterial drug development.

In order to eliminate the disadvantages of biomaterials used as raw materials for the production of medical devices, more and more attention has been paid to bio-composite structures. A bio-composite is a material consisting of two phases, at least one of which is bio-derived or biodegradable. The use of bio-composites brings many benefits, mainly in the context of their use as raw materials for medicinal products. The use of bio-composites also translates into a reduction in the use of “petroleum-derived” products. These materials have many environmental benefits by reducing CO_2_ emissions and ensuring sustainable waste management due to their recyclability [64].

#### 2.3.2. Synthetic Polymers

The use of synthetic polymers as templates and templates in bioengineering has several key advantages over naturally derived polymers, including options to control shape, architecture, and chemistry to generate reasonable alternatives or mimics of human-derived ECM systems that emulate or control the functions of biomaterials [65]. The most commonly used synthetic polymers for tissue regeneration are poly(hydroxy acids), which include polylactic acid (PLA), polyglycolic acid (PGA), and their copolymer, poly(lactic-co-glycolic acid) (PLGA) [66]. The non-toxic degradation products of these polymers (lactic acid and glycolic acid) are formed by simple chemical hydrolysis of the polymers and are removed by normal metabolic pathways [67]. Given the lack of dependence on local enzyme concentrations, chemical hydrolysis can be more easily predicted and controlled than enzymatic degradation in vivo [66]. The properties of synthetic polymers, such as tensile strength, mechanical modulus, and degradation rate, can be easily tailored to the intended applications by changing lactide/glycolide ratios and polymerization parameters.

Indeed, these materials have been successfully used in the clinic for the formation of urethral tissue as well as for bladder replacement in patients with idiopathic detrusor or neurogenic bladders [68,69,70,71]. In addition, in situ-forming hydrogels based on synthetic polymers can be engineered to locally deliver a wide range of bioactive agents in a controlled and sustainable manner to regulate the fate of stem cells encapsulated in a network of 3D polymers such as polyethylene glycol [72]. Due to its unique properties, such as biocompatibility, low immunogenicity, hydrolysis under physiological conditions, and approval by the Food and Drug Administration for clinical use, poly(-caprolactone) (PCL) is another synthetic polyester based on hydroxyalkanoic acids that have attracted a lot of interest in tissue engineering. This polymer is used alone, as a hydrophobic PCL, or as an amphiphilic PCL-containing block copolymer in combination with other agents, resulting in better performance in some applications [73,74,75]. Many synthetic polymers (e.g., PLGA, PEG, PCL, polyacrylic acid, polyvinyl alcohol, and polyvinylpyrrolidone) owe their widespread biomedical use to ECM-like biomimetic micro/nanoscale fibers, attractive processability, and biocompatibility. Although synthetic polymeric biomaterials can produce scaffolds with fully connected pores, some classes, such as poly(α-hydroxyesters), can produce acidic degradation products that can alter the pH of surrounding tissues [74]. In turn, this change in pH may affect cell behavior and survival and cause adverse tissue and inflammatory reactions [75].

Synthetic polymers themselves typically do not carry the risk of eliciting an immune response due to the lack of biologically functional domains. This feature is also a limitation as the lack of reactivity of the peptide side chain to bind regulatory peptides, growth factors, and other biological signals does not allow cell adhesion or direct phenotypic expression to be facilitated as a natural polymer would. However, various synthetic techniques have been developed and optimized to incorporate biologically active domains into synthetic polymer matrices, thus enabling the production of biomimetic scaffolds with a defined and regulated composition [76]. For example, synthetic polymer scaffolds coated with collagen or serum are usually sufficient to allow for initial cell attachment and ECM deposition, while ceramic (calcium phosphate or CaP) coating of synthetic polymer scaffolds is critical for bone engineering applications [77,78]. In other cases, synthetic polymeric scaffolds have been produced and modified by covalent immobilization of ECM-derived moieties to enable the spatio-temporal presentation of biological drugs, promote cell attachment, and enhance targeted differentiation of progenitor cell populations [78]. The presentation of bioactive agents on synthetic polymer matrix surfaces is the most effective way to induce the desired cell–material interactions. The ability to engineer these polymer systems to influence the behavior and interaction of cells is another key feature that provides both fundamental insight into the chemistry of structure–function relationships and great potential for the direct use of these biomaterials as cellular scaffolds [79].

The presence of such a wide range of synthetic polymers has made these materials promising for medical purposes. This is due not only to the ever-increasing supply from constantly developed technologies, such as tissue engineering, regenerative engineering, or nanotechnology, but also to the fact that synthetic materials have high repeatability and “certainty” of origin [80]. However, it should be remembered that the suitability of a given synthetic material for medical applications is conditioned by the structure of the main compound that is part of the polymer and the type of additional component used [4]. Currently, various synthetic polymeric materials are used in many medical applications, including polypropylene (PP), polyethylene (PE), polymethylmethacrylate (PMMA), polyethylene terephthalate (PET), and polyurethanes [35]. However, biodegradable polymers have recently gained a lot of interest. These materials, due to the fact that they have features such as biocompatibility and biodegradability, have become promising materials in medicine. This is due to the fact that the products that will be formed during the decomposition of this material will be absorbed or excreted by the body without causing any harm to it. Such materials include, among others: polyglycolide (PGA), polylactide (PLA), polycaprolactone (PCL), and their copolymers.

Polymer biomaterials are used, among others, as resorbable substrates for tissue regeneration, structural implants, cements, or biostable bonding elements and dressing materials. Thanks to their antimicrobial properties, polymers have been used as drug carriers. Polymeric materials are also used to reduce friction and corrosion, as dental adhesives, and to regenerate tooth pulp and dentin. In addition, polymer composites meet other requirements such as appropriate strength and biological properties, anti-corrosion behavior, easy availability, relatively easy processing, and low cost of production. In addition, the use of polymer coatings enables increased biocompatibility of bulk materials such as bio-ceramics [27].

Chitosan is a linear copolymer of β-(1–4) linked 2-acetamido-2-deoxy-β-d-glucopyranose and 2-amino-2-deoxy-β-d-glycopyranose [71]. The interest in chitosan structure and application dates back to the 19th century. In 1859, for the first time, Rouget discussed the deacetylated forms of the parent chitin natural polymer widely distributed in nature (2). Currently, chitosan is approved by generally recognized as safe (GRAS) by the U.S. Food and Drug Administration (FDA).

### 2.4. Discussion

Thanks to the ability to produce composites with specific physical, chemical, and mechanical properties, bio-composites are an ideal material for various medical applications. The most important features of bio-composites are their ability to biodegrade and biocompatibility with human tissue, which ensures the possibility of renewing damaged tissues. In addition, these materials have good mechanical properties and high resistance to corrosion and wear, which enables long and easy use of products made of this type of material. The properties of bio-composite materials can be adapted to their further use. By applying the simplest modifications of such parameters as mass ratios of components, fiber particle sizes, the geometry of the structure of additives as well as their orientation and distribution in the matrix, a composite with improved functional properties can be obtained. As a result, biomaterial composites show design flexibility and much better properties compared to metallic, ceramic, or polymer biomaterials [81,82,83,84]. However, it should be remembered that due to the formation of multi-phase compounds, bio-composites must be subjected to many biological tests before they can be potentially used for the production of medical devices.

**Figure 5:**
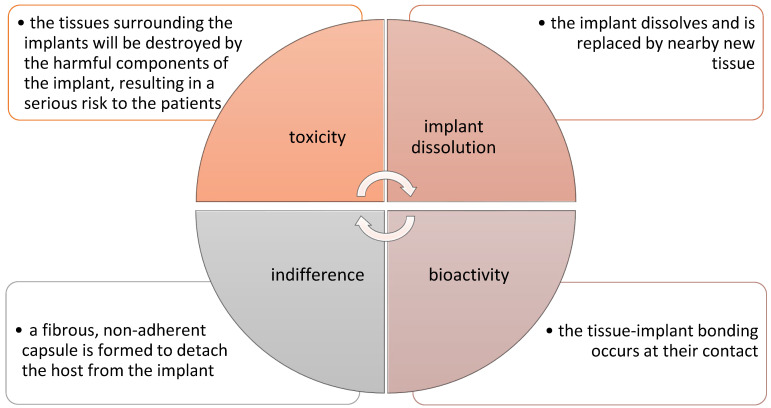
Various implant–tissue interactions and their reactions inside the body [86].

Currently, numerous studies are being carried out on the use of polymer–ceramic composites as a material in the regenerative medicine of bone tissue. This approach allows for a more accurate reconstruction of the entire bone structure. Polymer matrices combined with bio-ceramics or bioactive glasses allow imitation of the organic and mineral phase found in the bone, which enables better regeneration of damaged tissue or organ [85]. Depending on the application, polymer–ceramic composites must have specific characteristics. In the case of using this type of material for the regeneration of bone tissue, the extremely important features are biocompatibility, biodegradation, appropriate microstructure, as well as optimal distribution and pore size for good connection of these materials with cells. In addition, the material must have good fatigue strength, high wear, and corrosion resistance. In general, four types of implant–tissue interactions must be met in the body after surgery for proper function and regeneration of damaged tissue. Various implant–tissue interactions and their reactions inside the human body are shown in Figure 5 [86].

There are many articles in the literature concerning the combination of biodegradable polymers with hydroxyapatite (HAp). The combination of these two biomaterials makes it possible to obtain composite scaffolds that have high bioactive and osteo-inductive abilities [87,88]. Hydroxyapatite with the formula Ca_10_(PO_4_)_6_(OH)_2_ is a compound derived from the group of calcium phosphates, with a molar ratio of 1.67 Ca/P. This biomaterial is used for various biomedical applications, mainly as a material in tissue engineering, but also as a component of polymer matrices used in the production of drug release control systems. Pure hydroxyapatite is a brittle material with low flexibility and hardness, which makes its strength properties too low to be used alone in tissue engineering.

Due to its mechanical properties, hydroxyapatite can be used as a composite matrix or as its filler for the following reasons [89,90,91,92]:

- It is similar in structure and composition to natural bones and teeth, and it improves the biocompatibility of bio-composites;
- By introducing HAp particles into a polymer matrix, it is possible to improve the degradation rate of composites due to the bioactivity of HAp.

Hydroxyapatite additives can be present in various forms, e.g., as particles, fibrils, rods, whiskers, or nanoparticles [87]. Particles and nanoparticles of hydroxyapatite (n-HAp) are currently very often used as fillers for the production of bone scaffolds based on biodegradable metals and polymers. The presence of HAp in a polymer matrix affects the adsorption of proteins and the adhesion of bone cells, and the use of fillers in the form of n-HAp with a high aspect ratio can improve the mechanical properties of polymer-based composites [93,94].

Recently, hydroxyapatite is a widely considered material for the production of metallic implants based on biodegradable metals, such as magnesium (Mg). Many studies have shown that the introduction of hydroxyapatite to the magnesium matrix can improve the biocompatibility, mechanical properties, and corrosion resistance of the obtained products [95]. Researchers from Portugal have recently developed a biodegradable magnesium-based composite with the addition of hydroxyapatite nanoparticles and fluoroapatite (FA) microparticles for medical applications. The addition of the fillers used was intended to improve the bioactivity and compatibility of magnesium. This new innovative composite was produced using the Friction Up Mixing (UFSP) method. The technology they used, consisting of grinding magnesium grains, is a very well-known technique for obtaining metal matrix composites. The ceramic particles introduced into the magnesium matrix were obtained by using the hydrothermal method supported by citric acid. By examining the structure of the obtained composites, it was proved that the HAp and FA particles are well dispersed in the matrix, and the Mg grain size significantly decreased after the UFSP process. The obtained Mg/HAP/FA and Mg/HAP composites were tested in vivo for their bioactivity in simulated body fluids. The results showed that a layer of fluoride-rich apatite had formed in the composite, which was attributed to the release of fluoride ions from the composite and their precipitation in different configurations. Moreover, the compatibility results showed that the presence of FA particles together with HAp nanoparticles may promote the interaction of osteoblasts with the biomaterial. Therefore, obtaining a Mg/HAp/FA composite with such satisfactory properties can be considered a promising material for orthopedic applications [96].

The use of a filler in the form of hydroxyapatite resulted in obtaining a PLA/HAp composite with high biocompatibility and high cell viability in vivo. The addition of apatite fillers also ensured the neutralization of by-products formed during the decomposition of polylactide, which prevents the formation of inflammation in the body [87,97]. The PLA/HAp composite, due to its outstanding properties, has become a potential material for medical applications, mainly in areas such as tissue and regenerative engineering. In 2021, a group of scientists from Japan conducted research on the use of PLA/HAp composite as a carrier in drug delivery systems. In their research, they prepared a shell in the form of the considered composite by using the emulsion method. As a model drug, they used vitamin K1 due to its ability to dissolve in fats. The prepared samples were tested for drug loading and drug release in phosphate buffer solutions. Drug loading studies have shown that as the amount of drug injected increases, the particle size also increases, proving that the shell is working properly. During tests of the release of vitamin K1 in a buffer solution, it was proved that the amount of the released vitamin increased with a decrease in the pH of the buffer, due to the fact that the HAp coating easily dissolved under acidic conditions. PLA/HAp particles have been found to be promising candidates as drug delivery vehicles due to their excellent drug-loading capacity and pH sensitivity [98,99].

Due to the favorable properties of biodegradable polymers for medical applications, their copolymers are advantageous variants that make it possible to exclude their unfavorable disadvantages. Over the years, a wide variety of copolymers have been explored for bone repair applications, with polylactide/polyglycolide copolymer becoming a promising material. In the paper, Hassan and other researchers developed a new bioactive, porous scaffold based on the aforementioned copolymer. They developed a composite based on poly(lactide-co-glycol) (PLGA) with the addition of nano-hydroxyapatite with an admixture of strontium and zinc (Sr/Zn n-HAp). The obtained test results of this innovative composite showed that the developed material showed high porosity, and its bioactivity was proven by immersing the samples in a simulated body fluid. In addition, it was shown that the composite immersed in body fluid for a week had Sr, Ca, and Zn ions in its structure, which in the case of implantation would increase the degree of osseointegration. The material also had good strength and biodegradable properties. One of the developed composites containing 2.5% Sr/Zn admixture exhibited a compression behavior similar to that of bone tissue. The authors of the article announced further research into the use of this material for the production of bone implants [100,101].

In recent years, numerous studies have been conducted on modifications of PLA/HAp composites in order to accelerate the degradation of the composite scaffold. In their research, researchers from China developed a composite that additionally contained poly(glycolic acid) (PGA), due to its rapid degradation rate. Composite scaffolding was made using 3D laser printing. The obtained results showed that the inclusion of PGA increased the rate of degradation, as demonstrated by the increasing weight loss of the samples after one month of immersion in the PBS solution. This was due to the rapid degradation of the highly hydrophilic PGA and the subsequent accelerated hydrolysis of the PLLA chains. In addition, more pores were formed during degradation, which had a positive effect on the cell culture rate. The developed PLLA/PGA/HAp composite turned out to be a material with the desired biodegradable and biocompatible properties with human tissue, which gives this material high potential for implantology applications, especially for the production of highly porous bone scaffolds [102].

In order to improve the connectivity between the tissue and implants made of PCL, hydroxyapatite was introduced into the polymer matrix. The introduction of an additive in the form of bio-ceramics made it possible to improve the mechanical properties and compatibility of the obtained products. In one of the articles from 2021, Montloung and coworkers examined how the reduction in the HAp content in the composite affects the properties of PCL and its further use in the industry. The composite was prepared by using the melt-mixing technique, as this method is widely used in the industry. In the studies carried out, the amount of hydroxyapatite used varied from 1 to 7% by weight, which is a very small amount considering that in studies relating to medical applications, the amount of hydroxyapatite is up to 60%. It was shown that hydroxyapatite disperses much better at low concentrations in PCL, while agglomerations could be seen at higher contents of this additive. The mechanical properties of the obtained products, compared to the reference sample, improved with the increase in the hydroxyapatite content, although the researchers assumed their decrease due to the possibility of agglomeration in the composite. In addition, a significant decrease in crystallinity was observed with increasing bio-ceramic content, which may have affected the strength properties. The conducted research showed that the developed composites can be used for various applications, including the production of foams and foils for packaging applications [103].

In 2021, researchers from Poland developed a three-component composite that can be used to produce bone scaffolds. The composite was developed on the basis of poly(ε-caprolactone), hydroxyapatite whiskers, and L-lysine (Lys) being used as a filler. The scaffolds were made using the thermal alloy induction technique (TIPS). This technique makes it possible to modify the pore morphology in a simple way by determining the appropriate thermodynamic parameters of the process. The resulting composite had a porosity of more than 90% and a much higher Young’s modulus than the PLA/HAp reference sample. By introducing L-lysine, the compressive strength was significantly improved. Researchers have shown that the resulting composite allows cell growth. In addition, the composite showed better biocompatibility compared to other PCL-based scaffolds, which means that the obtained three-component composite can be considered a good candidate for the production of biomedical devices [104].

In recent years, polyetheretherketone (PEEK) has also enjoyed great interest for medical applications. It is a high-quality material with high crystallinity, resistance to high-energy radiation, excellent thermal stability, and resistance to most substances except concentrated sulfuric acid. This material has very good mechanical properties, which do not change during sterilization using steam, gamma radiation, or ethylene oxide [105]. In addition, PEEK has tensile strength properties similar to human bone and does not show any mutagenic or cytotoxic activity. Polyetheretherketone is resistant to degradation, which makes it a widely desired material in such fields as biomedicine, aviation, and automotive [106].

Polyetheretherketone has a hydrophobic character, which limits cell adhesion and protein absorption, and the process of modifying this material has begun. The conducted studies have shown that the biocompatibility of PEEK can be improved by introducing such additives as carbon fibers, Ti, TiO_2_, or MgO particles into its structure [107]. However, for the production of implants intended to bond to bone tissue, the most preferred additive is hydroxyapatite. The combination of PEEK with HAp ensures obtaining a product with a similar stiffness to bone tissue. The conducted research showed that the incorporation of hydroxyapatite particles optimized the bioactivity and mechanical properties of this material. A group of researchers from India recently developed a synthetic membrane consisting of PEEK and HAp nanoparticles. This material has been tested for its use in the manufacture of hip bone implants. In addition, researchers proposed a new approach to the synthesis of the developed membrane. In order to improve the interactions between the polymer and bio-ceramics, PEEK was subjected to sulfonation, which probably can reduce the resorption of the implant made of PEEK/HAp material in the body. Better surface wetting, obtained by sulfonation of the matrix, facilitated the reinforcement of the matrix with HAp nanoparticles. The obtained composites also had better mechanical properties, which means that the membranes designed in this way may become good candidates for the production of hip bone implants [108].

The introduction of bioactive nanofillers and the creation of porous surfaces are currently two commonly used modifications to improve the compatibility of PEEK with cells. Huang and his research group conducted research on the simultaneous implementation of both of the mentioned PEEK modification strategies. Researchers introduced nanoparticles of graphene oxide (GO) and hydroxyapatite into the polymer matrix. The three-component composite was obtained by extrusion and injection molding, followed by laser treatment, in order to obtain macropores with diameters of 200–60 μm on the surface of the product. The structural results showed that pores with a depth of 50 μm were formed on the surface of the composites, which did not significantly affect the mechanical properties of the composites obtained. However, the introduction of GO and HAp nanoparticles improved cell adhesion and proliferation on the PEEK surface. The obtained research results show that the use of nanofillers and macropores on the surface of composites can be a promising way to improve the tissue integration of PEEK in bone implants [109].

## 3. Materials and Methods

This review was conducted through databases such as PubMed, Google Scholar, and Elsevier. The type of publications considered included reviews and articles in the English language were mainly analyzed. The PICO (population, intervention, control, and outcomes) criteria are included in the format of Table 1.

**Figure 6:**
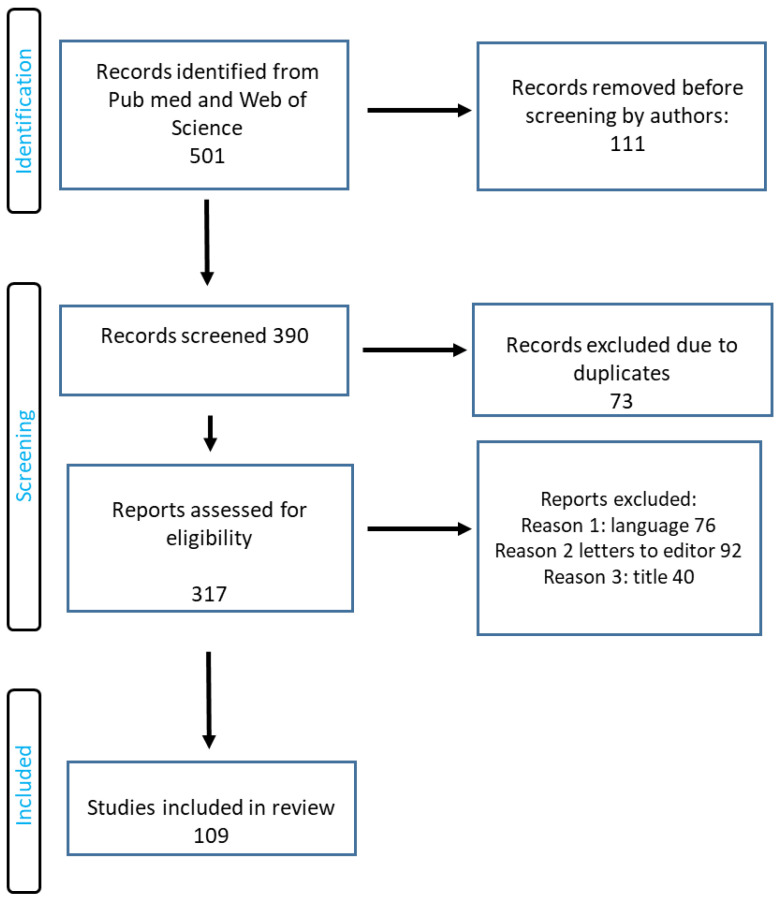
RISMA flow diagram of included studies.

The search term included the phrases “biodegradable polymers”, “biomaterials”, “applications of biomaterials in medicine”, and “applications of biodegradable polymers in medicine”. The authors of this review worked on the basis of an agreed scheme, selecting articles based on their title, language, abstract, and access. Duplicate records were removed. PRISMA flow diagram is presented in Figure 6.

## 4. Conclusions

Currently, numerous studies are being carried out on the use of polymer–ceramic composites as a material in the regenerative medicine of bone tissue. This approach allows for a more accurate reconstruction of the entire bone structure. Polymer matrices combined with bio-ceramics or bioactive glasses allow imitation of the organic and mineral phase found in the bone, which enables better regeneration of the damaged tissue or organ. Depending on the application, polymer–ceramic composites must have specific characteristics. In the case of using this type of material for the regeneration of bone tissue, the extremely important features are biocompatibility, biodegradation, appropriate microstructure, as well as optimal distribution and pore size for good connection of these materials with cells.

## Figures and Tables

**Table 1:** The research question defined as PICO criteria.

PICO component	Abstract component inherent to all research designs
Problem	Research object: biodegradable polymers and biomaterials
Intervention	Application of a theory or method; biocompatibility, appropriate mechanical properties, ease of sterilization, high porosity, ensure an improvement of living
Comparison	Alternative theories or methods (or, in their absence, the null hypothesis); specimens did not receive surface conditioning before bonding
Outcome	Knowledge generation: medical, dental or esthetical application.

## Data Availability

All data have been included.
